# The pencil eraser swab technique to quantify *Cutibacterium acnes* on shoulder skin

**DOI:** 10.5194/jbji-6-451-2021

**Published:** 2021-12-17

**Authors:** Vendela M. Scheer, Malin Bergman Jungeström, Lena Serrander, Johan H. Scheer, Anders Kalén

**Affiliations:** 1 Department of Biomedical and Clinical Sciences, Linköping University, Linköping, 581 85, Sweden; 2 Faculty of Health Sciences, Linköping University Hospital, Linköping, 581 85, Sweden; 3 Division of Clinical Microbiology, Department of Biomedical and Clinical Sciences, Linköping University, Linköping, 581 85, Sweden; 4 Division of Orthopedics, Department of Biomedical and Clinical Sciences, Linköping, 581 85, Sweden

## Abstract

**Introduction**: *Cutibacterium acnes* is the most common cause of postoperative infections in orthopaedic shoulder surgery and is hard to eradicate with current measures. Newer strategies focus on reducing bacterial load on the skin before surgery. Several previous studies have used a large number of both described and undescribed sampling techniques. The purpose of this study was to compare three previously described swab techniques to obtain bacterial cultures: Levine's (L) technique, the Z technique and the pencil eraser swab (PES) technique. **Methods**: Three consecutive skin swabs were collected from the right shoulder, on 15 healthy male volunteers, using Levine's technique, Z technique and PES technique from each participant. To determine the number of living bacteria, serial dilutions were made, and after culturing for 5 d, viable count (VC) was expressed as CFU/mL (with CFU representing colony-forming unit). **Results**: The PES technique yielded significantly higher VC than the two others. PES: median 3700 CFU/mL, L: 200 CFU/mL and Z: 220 CFU/mL ( p=0.003 ). There was no significant difference between the methods regarding the number of positive cultures. PES: 14/15, L: 11/15 and Z: 12/15. **Conclusions**: There is a need to harmonise sampling techniques of *C. acnes* in order to compare the efficacy of different measures to reduce the bacterial load on the skin before and during surgery. Of the three tested methods, the PES technique is simple and produces the highest bacterial counts.

## 1 Introduction

In orthopaedic surgery, surgical site infections (SSIs) are usually caused by the patient's own skin flora, so-called endogenous infection (Krizek and Robson, 1975). *Cutibacterium acnes* (*C. acnes*) resides in the sebaceous glands of the skin and are the most common bacteria causing SSI after orthopaedic shoulder surgery (Achermann et al., 2014; Levy et al., 2008; Nelson et al., 2016; Richards et al., 2014). Earlier studies have demonstrated that this species can prevail on the skin despite strict preoperative preparation with alcohol-based chlorhexidine (Lee et al., 2014; Scheer et al., 2021). This has spawned investigation of other eradication strategies, one being to evaluate whether different bactericidal creams applied before surgery can reduce the number of bacteria on the skin (Chalmers et al., 2019; Dizay et al., 2017; Hancock et al., 2018; Murray et al., 2011; Sabetta et al., 2015; Stull et al., 2020) with the presumption that this in turn will reduce bacterial load in the surgical field. Since SSIs in open orthopaedic surgery are relatively rare events (0.3 %–5.0 %) (Singh et al., 2012; Padegimas et al., 2015; Atesok et al., 2017; Eck et al., 2018), showing actual reduction of infection frequency requires tens of thousands of patients in huge multicentre trials. Therefore, commonly, bacterial skin count is indirectly used as an assessment of the effectiveness of a method (Falk-Brynhildsen et al., 2013b; Chalmers et al., 2019; Meyer et al., 2021).

Studies on the subject use different bacterial sampling methods. Several papers use their own, previously non-described method (Falk-Brynhildsen et al., 2013a; MacNiven et al., 2018; Blonna et al., 2018), and unfortunately a large number of studies on the subject do not even described what sampling technique is used (Egli-Gany et al., 2012; Murray et al., 2011; Dizay et al., 2017; Matsen et al., 2013; Chuang et al., 2015; Sethi et al., 2015), which makes comparison of results difficult. This is illustrated by the fact that dermal colonisation of *C. acnes* on normal untreated skin in different studies varies between 30 %–97 % (Dizay et al., 2017; Kolakowski et al., 2018; Sabetta et al., 2015; Scheer et al., 2021).

The three most described and used methods for bacterial skin sampling are Levine's technique (Levine et al., 1976), the Z technique (Angel E Donna et al., 2011) and the swab-cup technique (Williamson and Kligman, 1965). The first two were developed to obtain cultures for diagnosing wound infections in clinical practise. The third is not a swab technique in the same sense since a swab is used to stir up a solution in a cylinder held against the skin, and the solution is then aspirated for culture.

We have in two previous studies (Scheer et al., 2018, 2021) introduced yet another method, the PES technique (pencil eraser swab) after executing a number of pilot studies (using chambers, scrapes, changing the number of swab passages, etc.) with the aim to maximise the number of positive cultures with *C. acnes*. The purpose of this study was to compare the PES method to Levine's technique and the Z technique. This was done with the hypothesis being that the PES technique would yield more positive *C. acnes* cultures and a higher bacterial count as compared to the other two techniques. The swab-cup technique was not investigated since it is too laborious to use in a surgical setting and has produced low viable bacterial counts of anaerobic bacteria (Dorfel et al., 2021).

## 2 Materials and methods

Fifteen healthy male volunteers consented to undergo culture of three skin swabs on unprepared skin. Previous studies have shown that men have more *C. acnes* than women; hence, we included men only (Patel et al., 2009; Dizay et al., 2017; Scheer et al., 2021). Healthy hospital staff members were asked to participate in the study. Written informed consent was obtained from all participants. Inclusion criteria were male, age >18 and with legal capacity. Exclusion criteria were any visible skin lesion in the shoulder area or any antimicrobial treatment within 7 d of the swabbing (to maximise bacterial count).

**Figure 1:**
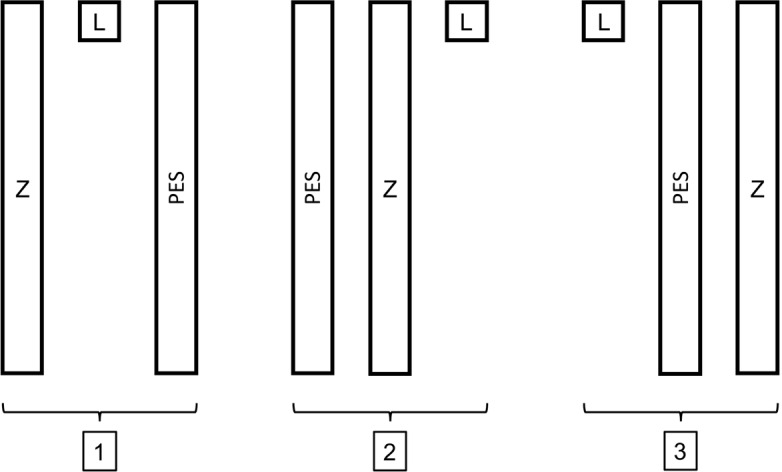
The three different templates. Z: Z technique, L: Levine's technique and PES: pencil eraser swab technique. Each rectangle has the dimensions of 1cm×10 cm, the square measures 1cm×1 cm, and there is 1 cm between each template.

Three consecutive skin swabs were collected from the right shoulder, using the L technique, Z technique and PES technique from each participant. Three different sterile templates, nos. 1, 2 or 3, were used to ensure equal swabbing areas in all subjects (Fig. 1). The template number used for each volunteer was chosen by randomly selecting it from an envelope. For swabbing, we use eSwab (Copan Italia S.p.A. via Perotti 10, Brescia, Italy), a flocked swab with a tube, containing 1 mL of liquid Amies, which elutes the entire sample into the medium.

### 2.1 Sample techniques

**Figure 2:**
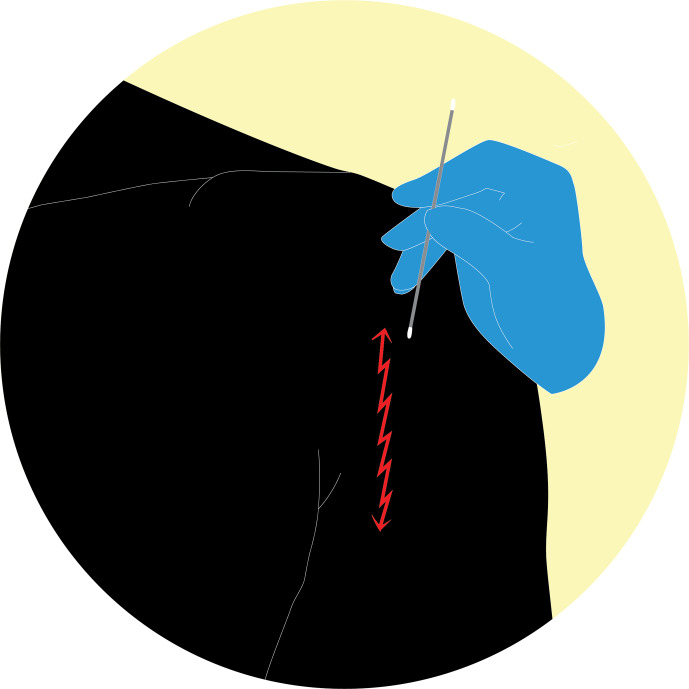
PES technique: rub the swab with an oscillating movement – like using a pencil eraser – going down over a 10 cm line and then in the same manner up again for a total of 15 passages (https://doi.org/10.5446/55554; Scheer, 2021a).

The Z technique involves rotating the swab in a 10-point zigzag fashion once – in this study over a 10 cm line, corresponding to a standard shoulder incision. Levine's technique (L technique) consists of rotating the swab over a 1 cm area with “sufficient” pressure for 5 s. Finally, the PES technique: rub the swab with an oscillating movement – like using a pencil eraser – going down over a 10 cm line and then in the same manner up again for a total of 15 passages (Fig. 2).

**Table 1:** Qualitative results; subjects with positive bacterial cultures ( n=15 ).

Bacteria	PES technique	Levine's technique	Z technique
*C. acnes* (no. of positive cultures)	14/15	11/15	12/15
CoNS (no. of positive cultures)	11/15	9/15	8/15
Median viable count [CFU/mL] (Range)	3700 (140–133 000)	200 (0–6600)	220 (0–4300)

*C.*: *Cutibacterium*, CoNS: coagulase-negative staphylococcus

### 2.2 Microbiological technique

All skin swabs were immediately put into the medium and within 1 h, transported to the laboratory; they were vortexed for 10 s whereafter serial dilutions were made, and these were cultured on anaerobic agar medium without antibiotics and placed in an anaerobic incubator. After 5 d, we counted the colony-forming units (CFU), and viable count (VC) is expressed as CFU/mL (Ben-David and Davidson, 2014). Bacteria species were detected with matrix-assisted laser desorption/ionisation time-of-flight mass spectrometry (MALDI-TOF).

### 2.3 Statistical analyses

Patzer, Phadnis and Falk-Brynhildsen (Patzer et al., 2018; Phadnis et al., 2016; Falk-Brynhildsen et al., 2013a) all used different swab techniques that yielded on average 42 % positive cultures of *C. acnes*. In two previous studies, using the PES technique, we had 97 % positive cultures of *C. acnes* (Scheer et al., 2018, 2021). With an 80 % power and a significance level of 0.05, a sample size of 15 subjects was required. We used the chi-square test for categorial variables. The Kruskal–Wallis test for ranks (one-way ANOVA on ranks) was used for comparing distributions with a following pairwise comparison with adjustment by the Bonferroni correction for multiple tests.

**Table 2:** Quantitative results in pairwise comparisons.

Pairwise comparison	Significance	Adjusted
between groups	level	significance*
L and Z techniques	0.813	1.00
L and PES techniques	0.004	0.013
Z and PES techniques	0.002	0.006

* Adjustment by the Bonferroni correction for multiple tests

## 3 Results

Fifteen male volunteers were enrolled in this study. Their average age was 46 (range 28–65). There were no significant differences between the different techniques in detecting *C. acnes* (positive cultures) or CoNS (coagulase-negative staphylococci) (Table 1). The CoNS found were identified as *Staphylococcus epidermidis*, *S. saccarolyticus*, *S. hominis*, *S. cristatus* and *S. capitis*. The one-way ANOVA on ranks showed that the distribution of VC was not the same across categories of groups ( p=0.003 ). The pairwise comparisons are displayed in Table 2 demonstrating the PES technique producing significantly higher viable counts with no difference between the Z and L techniques. In all techniques, 84 %–87 % of the CFUs were *C. acnes*.

**Table 3:** Positive *C. acnes* cultures in different studies.

Primary study	No. of	Male	Female	Positive *C. acnes* cultures	Swab
	patients	(%)	(%)	before treatment	technique
Chuang et al. (2015)	51	74	26	72 %	ND
Phadnis et al. (2016)	50	60	40	42 %	ND
Murray et al. (2011)	50	50	50	58 %	ND
Dizay et al. (2017)	65	66	34	48 %	ND
Matsen et al. (2013)	30	60	40	77 %	ND
Sabetta et al. (2015)	50	46	54	32 %	ND
Scheer et al. (2018)	40	60	40	95 %	PES
Scheer et al. (2021)	100	63	37	97 %	PES

ND: not described, PES: pencil eraser swab technique

## 4 Discussion

Our results suggest that the PES technique is effective in detecting high quantities of viable *C. acnes* compared to Levine's and the Z techniques. Hence, it may be more usable when evaluating measures to reduce bacterial load on the skin prior to surgery – at least for *C. acnes*.

When studying different aseptic preoperative preparations, it is important to have sensitive and reproducible methods. Results from earlier studies display considerable variation identifying *C. acnes* on the skin before preparation (Table 3). The true rate of *C. acnes* colonisation in the area is unknown, but we believe it is close to 100 % based on previous work on the microbiome of the skin (Huse et al., 2012). It is paramount that studies designed to evaluate preoperative preparation and its efficacy on reducing bacterial load describe the method transparently and completely. Even so, it is difficult to detect differences in preoperative techniques if the method in question has low sensitivity. Tape stripping, surface scrapes and cup-scrub technique are techniques that have been documented and validated for skin microbiome sampling (Kong et al., 2017; Chng et al., 2016). In clinical settings, the cup-scrub technique can be cumbersome to use, and the tape-stripping method may cause skin damage, making it unfit in a surgical setting. An optimal method must leave the skin uninjured by the sampling; otherwise, it could increase the risk of an SSI if the samples are taken perioperatively. The eSwab is inexpensive and the simplicity of the method makes it easy to use. Parada et al. (2018) point to the lack of consensus in prevention of shoulder arthroplasty infection in a survey and also that we need to create best practice guidelines to limit SSI after shoulder surgery.

### Limitations

The range of VC is large in all groups. Hopefully this reflects a difference in true bacterial load. The sampling was performed at different times during the day, but it was not noted in the protocol how long before the sampling that the subjects had showered. However, this would affect the VC in all techniques equally since all the sampling in each subject was performed at the same time. Also, comparison of VC was made statistically pairwise resulting in each subject acting as their own control unless there is a substantial local variation of the bacterial load within the 5cm×10 cm dimensions of the template. This seems unlikely but cannot be completely ruled out. It must be noted that we have sampled *C. acnes* on the skin when we really want to assess dermal bacterial load. We only presume that they correlate, but this has not, to our knowledge, been shown. It appears, however, highly likely that since *C. acnes* reside and thrive in the sebaceous glands and not superficially, a high skin count reflects a high dermal count.

We have used the PES method in a clinical study without obvious skin abrasion (Scheer et al., 2021), but we believe that rubbing with swabs with any method should be used with caution or not at all on delicate skin in conjunction with surgery in the area.

## 5 Conclusion

To our knowledge, this is the first study comparing different skin swab techniques on healthy skin. The PES technique is easy to use, appears effective in detecting *C. acnes* and gives a high bacterial yield. It could be used in future studies to evaluate preoperative measures.

## Supplement

10.5194/jbji-6-451-2021-supplement The supplement related to this article is available online at: https://doi.org/10.5194/jbji-6-451-2021-supplement.

## Data Availability

Underlying research data can be accessed by (Scheer, 2021b).

## Code availability

Data analyses were performed by using IBM SPSS Statistics.

## Video supplement

The video supplement related to this article is available online at (Scheer, 2021a).
