# Supplementary material for: The pencil eraser swab technique to quantify Cutibacterium acnes on shoulder skin
Source: J Bone Jt Infect. 2021 Dec 17;6(9):451–6. doi: 10.5194/jbji-6-451-2021 (PMC8738962; doi:10.5194/jbji-6-451-2021)
Supplement: The supplement related to this article is available online at: https://doi.org/10.5194/jbji-6-451-2021-supplement. [file jbji-6-451-supplement.zip › jbji-6-451-2021-supplement-title-page.pdf]

Supplement of J. Bone Joint Infect., 6, 451–456, 2021
https://doi.org/10.5194/jbji-6-451-2021-supplement
© Author(s) 2021. CC BY 4.0 License.

Supplement of

The pencil eraser swab technique to quantify *Cutibacterium acnes* on shoulder skin

Vendela M. Scheer et al.

Correspondence to: Vendela M. Scheer (vendela.scheer@liu.se)

- jbji-6-451-2021-supplement-title-page.pdf
- FullSizeRender.MOV

The copyright of individual parts of the supplement might differ from the article licence.
